# Whole-Genome Sequence of a Porcine Circovirus Type 2 Strain Detected in Assam, India

**DOI:** 10.1128/MRA.00593-21

**Published:** 2022-01-06

**Authors:** Arpita Bharali, Lukumoni Buragohain, Nagendra Nath Barman, Sophia M. Gogoi

**Affiliations:** a Department of Animal Biotechnology, College of Veterinary Science, Assam Agricultural University, Khanapara, Guwahati, Assam, India; b Department of Veterinary Microbiology, College of Veterinary Science, Assam Agricultural University, Khanapara, Guwahati, Assam, India; DOE Joint Genome Institute

## Abstract

Porcine circovirus-associated disease caused by porcine circovirus type 2 (PCV2) is a vital threat to the global pig industry. In this study, we have characterized the complete genome sequence of a PCV2 isolate, namely, Assam-01, belonging to the genotype PCV2d.

## ANNOUNCEMENT

Porcine circovirus type 2 (PCV2) is a common pig pathogen that is universally distributed and is found to be associated with several clinical manifestations, such as postweaning multisystemic wasting syndrome (PMWS), porcine dermatitis and nephropathy syndrome (PDNS), reproductive failure, and respiratory and enteric diseases, which are collectively known as porcine circovirus-associated disease (1–4).

PCV2 is a nonenveloped, single-stranded DNA (ssDNA) virus with a circular genome, belonging to the family *Circoviridae* (2, 4–6). The genome size of PCV2 is 1,766 to 1,769 nucleotides, with two major open reading frames (ORFs), namely, ORF1 and ORF2 (4). ORF1 encodes the proteins Rep and Rep′, whereas ORF2 (*cap* gene) encodes the capsid protein, the only structural protein and the most immunogenic protein (4, 7). Besides these, two other ORFs (ORF3 and ORF4) are also described in PCV2 (7). Based on *cap* gene sequences, eight genotypes of PCV2 (PCV2a to PCV2h) have been identified to date (8).

**FIG 1:**
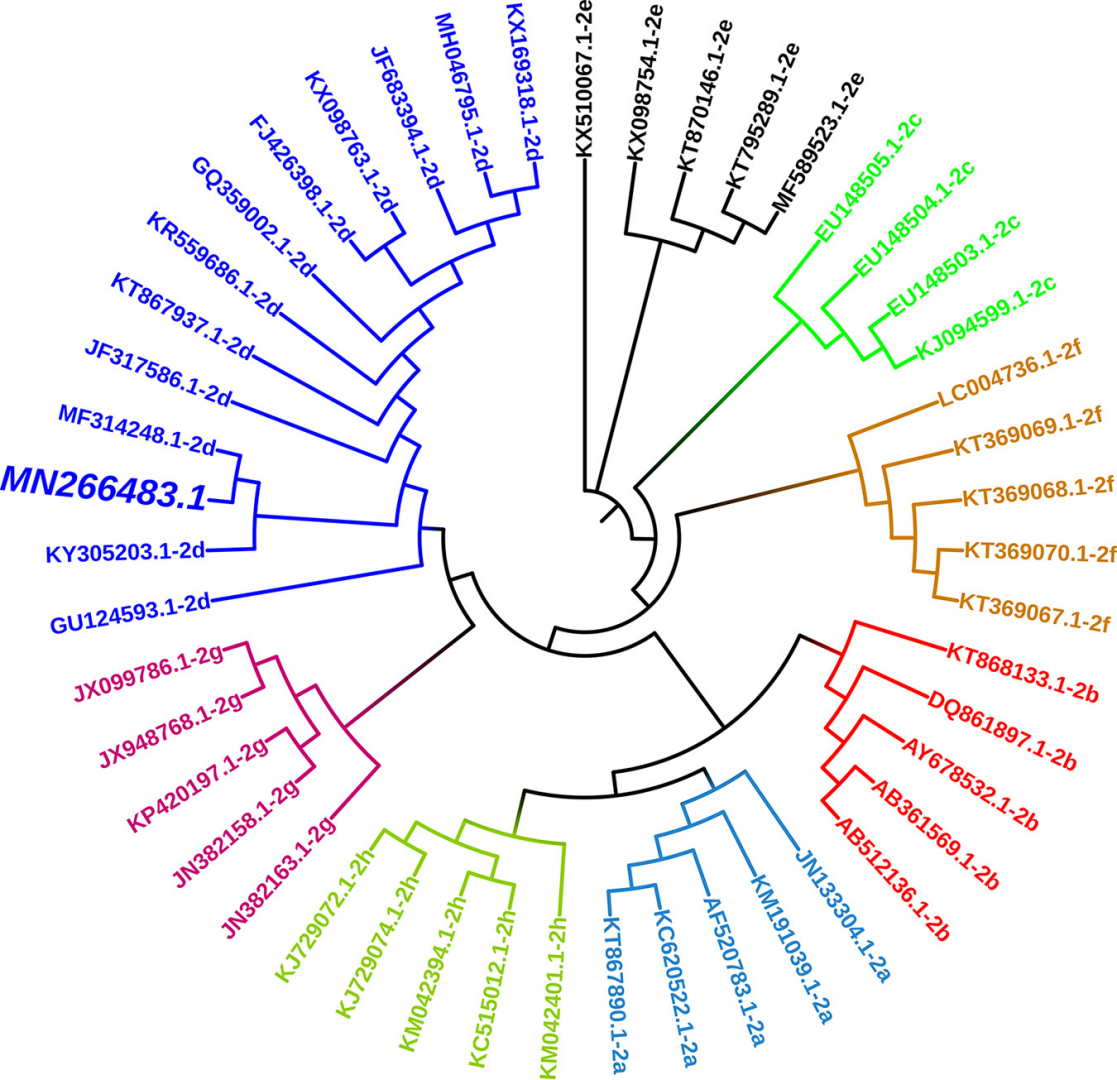
*cap* gene-based phylogenetic tree of PCV2. Multiple sequence alignment of *cap* gene sequences was performed by using the CLUSTALW program present in MEGA X software (12), and the phylogenetic tree was generated by the neighbor-joining (NJ) method (13) using the Tamura-Nei substitution model (14). The statistical reliability of the tree was evaluated with 1,000 bootstrap replicates. The tree was finally displayed using the online program iTOL (15). Clades with different colors represent eight different genotypes, along with their GenBank accession numbers and genotype names. The accession number of PCV2 isolate Assam-01 (accession number MN266483.1), which forms the clade with genotype PCV2d (dark blue), is highlighted in bold italic.

In the present study, the PCV2 isolate Assam-01 was isolated from a PCV2-PCR-positive pig tissue sample that is available in the repository of the Advanced Animal Disease Diagnosis and Management Consortium (ADMaC) Laboratory, College of Veterinary Science (Guwahati, India), and was received from a pig farm in Hekera, Kamrup, Assam, India, in 2017. The virus was isolated from pooled tissues (lung, liver, spleen, and kidney) from the same pig in PCV-free PK-15 cells by the method described earlier (9). The cells were harvested after the fourth passage, and DNA was extracted by using DNeasy blood and tissue kits (Qiagen). The complete genome of PCV2 isolate Assam-01 was amplified by PCR using two sets of overlapping primers (Table 1) that encompass the entire circular genome (10). The PCR products were purified, and the amplicons were subsequently sequenced by Sanger’s method, which was outsourced (1st BASE, Malaysia). The complete genome sequence of isolate Assam-01 was deduced and analyzed by using the online BLASTn tool (11) and BioEdit software. Phylogenetic analysis was performed with MEGA X (12) using *cap* gene nucleotide sequences retrieved from the GenBank database on 5 April 2021, representing eight genotypes of PCV2, as reported earlier (8). Sequence analysis revealed that the genome of PCV2 isolate Assam-01 was composed of 1,767 nucleotides, with a GC content of ∼48.4%. ORF1 was 945 nucleotides in length, encoding a protein of 314 amino acids, whereas ORF2 was 705 nucleotides in length, encoding the capsid protein with 234 amino acids. BLASTn results for Assam-01 showed the greatest identity (99.72%) with Chinese strains GXNN1 and DF-1. Phylogenetic analysis based on *cap* gene nucleotide sequences revealed that Assam-01 belongs to the genotype PCV2d (Fig. 1). The *cap* gene of PCV2 isolate Assam-01 exhibited 96.2 to 99.6% identity at the nucleotide level with other strains of the PCV2d genotype.

**TABLE 1:** Primer sequences used for amplification of the complete PCV2 genome (10)

Primer name	Primer sequence	Product size (bp)
CV1	5′-AGGGCTGTGGCCTTTGTTAC-3′	989
CV2	5′-TCTTCCAATCACGCTTCTGC-3′	
CV3	5′-TGGTGACCGTTGCAGAGCAG-3′	1,092
CV4	5′-TGGGCGGTGGACATGATGAG-3′	

Several studies have documented the worldwide prevalence of the PCV2d genotype, and India is no exception. Hence, this study could give better insight into the genomic diversity of PCV2 strains circulating in India, which is a prerequisite to formulating potential indigenous-strain vaccines in the future.

### Data availability.

The complete genome sequence of PCV2 isolate Assam-01 has been deposited in GenBank under the accession number MN266483.
